# Recurrent retroperitoneal abscess after biliary tract surgery in an elderly patient: a minimally invasive nonsurgical approach and its consequences: a case report

**DOI:** 10.1186/s13256-019-1973-3

**Published:** 2019-02-25

**Authors:** Vincenzo Davide Palumbo, Benedetto Di Trapani, Antonio Bruno, Mario Feo, Bernardo Molinelli, Simone Tomasini, Attilio Ignazio Lo Monte, Marianna Messina, Giovanni Tomasello

**Affiliations:** 1 Euro-Mediterranean Institute of Science and Technology (IEMEST), Via Emerico Amari, 123, 90139 Palermo, Italy; 2 Department of Surgical, Oncological and Stomatological Disciplines, University of Palermo, Palermo, Italy; 3 Casa di Cura Torina, Palermo, Italy; 4 Department of Diagnostic and Preventive Medicine, University of Bologna, Sant’Orsola, Malpighi Hospital, Bologna, Italy; 5 School of Medicine, University of Palermo, Palermo, Italy; 6 Department of Experimental Biomedicine and Clinical Neuroscience, University of Palermo, Palermo, Italy

**Keywords:** Case report, Hepatic abscess, Interventional radiology, Lumbar hernia, Minimally invasive procedures, Nonsurgical drainage

## Abstract

**Introduction:**

Hepatic abscess can be defined as an encapsulated collection of suppurative material within the liver parenchyma. Hepatic abscess can be distinguished as pyogenic, amebic, or fungal. Biliary tract disease remains the most common cause of hepatic abscess today, and the most common complications range from pleural effusion, empyema, and bronchohepatic fistula to subphrenic abscess and rupture into the peritoneal cavity, stomach, colon, vena cava, or kidney. A large abscess compressing the inferior vena cava and the hepatic veins may result in Budd-Chiari syndrome. In this report, we present a rare case of hepatic abscess with an unusual evolution that was treated with a noninvasive approach.

**Case presentation:**

A 79-year-old Caucasian woman underwent endoscopic bile stone extraction and laparoscopic cholecystectomy. Six months later, a hepatic abscess in association with bilateral effusion was diagnosed. The prompt imaging-guided drainage solved the case. Three years later, she came to our attention complaining of dull, diffuse abdominal pain and high body temperature (38 °C). A retroperitoneal abscess was diagnosed that was spreading to the right lateral wall of the abdomen and extending across the muscular wall to the subcutaneous layer. The fluid collection also involved the right pleural cavity, forming an empyema. Also in this case, an imaging-guided drainage was performed, and the patient’s clinical picture resolved in a few days. The retroperitoneal abscess recurred 14 months later, and it was dealt with using the same treatment. Three months from the last follow-up, the patient came back to our attention with an evident swelling of her right lumbar region. Computed tomography revealed a right inferior lumbar hernia comprising adipose tissue and the right kidney. A surgical intervention was recommended to the patient, but, owing to her poor general health, she refused any invasive approach.

**Conclusions:**

Retroperitoneal abscess is an uncommon complication of biliary tract surgery and represents a potential cause of death, especially in those patients with multiple diseases. Prompt drainage is crucial to the treatment. Failure in eliminating the primary infective focus could bring complications and, in general, a weakness of lumbar muscular wall, even resulting in a rare case of lumbar hernia.

## Introduction

Hepatic abscess (HA) can be defined as an encapsulated collection of suppurative material within the liver parenchyma. The three major forms of liver abscess, classified by etiology, are as follows:

- Pyogenic abscess, which is most often polymicrobial, accounts for 80% of HA cases in the United States.
- Amebic abscess due to *Entamoeba histolytica* accounts for 10% of cases [1].
- Fungal abscess, most often due to *Candida* species, accounts for less than 10% of cases.

Biliary tract disease remains the most common cause of HA today [2–5]. More recently, there has been an increase in the incidence of HA arising in association with malignancies and their treatment, including HA from liver metastasis [6, 7] and as a complication of transarterial chemoembolization or radiofrequency ablation [8–12]. Although the frequency of HA varies by region [13], the overall incidence is fairly low, ranging from 2.3 cases per 100,000 hospital admissions in North America [13] to 275.4 per 100,000 in Taiwan [14]. In the early 1900s, mortality was as high as 75–80% [6]; today, mortality is markedly decreased, ranging from 10% to 40% [5]. This is due to improvements in antibiotic therapy and interventional procedures for the treatment of HA [3, 6, 15]. The complications of HA result from rupture of the abscess into adjacent organs or body cavities. They may be broadly divided into pleuropulmonary and intra-abdominal types. Pleuropulmonary complications are the most common and have been reported in 15–20% of early series. These include pleurisy and pleural effusion, empyema, and bronchohepatic fistula [16]. Intra-abdominal complications are also common. They include subphrenic abscess and rupture into the peritoneal cavity, stomach, colon, vena cava, or kidney. A large abscess compressing the inferior vena cava and the hepatic veins may result in Budd-Chiari syndrome. Rupture into the pericardium or brain abscess from hematogenous spread is rare.

In this report, we present a rare case of lumbar hernia caused by a recurrent HA. A noninvasive imaging-guided approach was crucial to definitively cure the initial clinical picture, although it could not avoid the long-term consequences of a retroperitoneal purulent collection. This work is reported in line with the consensus-based guidelines for surgical case reports criteria [17].

## Case presentation

A 79-year-old Caucasian woman came to our attention complaining of dull, diffuse abdominal pain and high body temperature (38 °C). She had high blood pressure and type 2 diabetes, for which she was receiving oral ramipril 5 mg twice daily and metformin 500 mg three times daily, respectively. She was a housewife. Her parents had died of cardiovascular diseases in advanced age. She denied tobacco or illicit drug use and rarely drank a glass of wine. The patient reported a history of bile stones in her gallbladder and her common bile duct. She stated that she had undergone endoscopic bile stone extraction (endoscopic retrograde cholangiopancreatography with papillotomy) and laparoscopic cholecystectomy 3 years before. Cholecystectomy was completed with choledochotomy in order to extract further bile stones and for the positioning of a Kehr’s T tube. Postoperatively, a single daily dose of prulifloxacin 600 mg was taken for 5 days.

Two months later, the Kehr’s T tube was removed, and a new antibiotic therapy (always with single oral intake of prulifloxacin 600 mg for 5 days) was established. At reevaluation with abdominal computed tomography (CT), no bile leakage or biliary obstruction was detected.

Six months later, the patient returned to the hospital with a 4-day history of high body temperature (> 38 °C), right upper abdominal quadrant dull pain, and dyspnea. Her bowel sounds were normal; at palpation, abdomen was globally painful, with a mild tenderness at the right upper quadrant. Chest examination showed bilateral lower diaphragmatic excursion, decreased vocal fremitus, and attenuated sounds at pulmonary bases. The patient was oriented, and her language was fluent with good comprehension. Her neurological examination result was normal: Her pupils were equal, round, and reactive to light; visual fields were intact to confrontation; fundi were normal; ocular movements were intact; muscle bulk and tone were normal; sensation was intact to light touch, pinprick, vibration, and proprioception throughout; Romberg test result was negative; reflexes were normal throughout, and plantar response was flexor bilaterally; no dysmetria was observed on finger-nose-finger or heel-knee-shin; normal rapid alternating movements; and fast finger tapping with normal amplitude and speed. The patient’s blood pressure was 110/70 mmHg, and her heart rate was 97 beats per minute. Blood laboratory parameters showed severe leukocytosis (19,000 white blood cells/mm^3^). The patient was promptly started on broad-spectrum antibiotic therapy with endogenous piperacillin-tazobactam 4.5 mg twice daily and endovenous levofloxacin 500 mg once daily. An x-ray showed a homogeneous opacification of the right pulmonary lower zone and a slight elevation of the homolateral hemidiaphragm; the left costophrenic angle was obliterated with a meniscus. The findings were suggestive of a bilateral pleural effusion most relevant in the right side. Abdominal ultrasonography (US) showed multiple hypoechoic, loculated fluid collections within the liver parenchyma consistent with HA and confirmed pleural effusion. The subsequent contrast-enhanced chest and abdominal CT scan confirmed the presence of the HA (a 13-cm hypodense circular mass at hepatic segments VI, VII, and VIII) and marked the presence of a mild bilateral pleural effusion. Then, the patient underwent CT-guided drainage of the HA; at the same time, a 7-F multipurpose drainage catheter was positioned. In this case, the collected fluid was not examined. The chest and abdominal CT scanning performed 5 days later showed a severe right pleural effusion, whereas the left one remained superimposable to that shown 5 days before; the multipurpose drainage catheter was in the right position (close to the hepatic bare area), surrounded by plenty of retroperitoneal fluid.

The patient was discharged with oral antibiotics (levofloxacin 500 mg once daily and ceftriaxone 400 mg once daily for 15 days), and a control contrast-enhanced CT scan was scheduled for 1 month later. In this case, the examination showed a mild right pleural effusion and a well-circumscribed 9.5 × 4.5 × 9-cm fluid collection in the back of the thoracic cavity, pushing against the lower lobe of the right lung; abdominal slices showed a small 15-mm formation, laying on the posterior surface of the liver and imprinting the VIth segment, resulting from the drained abscess.

The subsequent control chest and abdominal CT scan, obtained 4 months later at the same hospital, showed an improved picture with a slight residual right pleural effusion and no signs of thoracic abscess or HA.

**Fig. 1:**
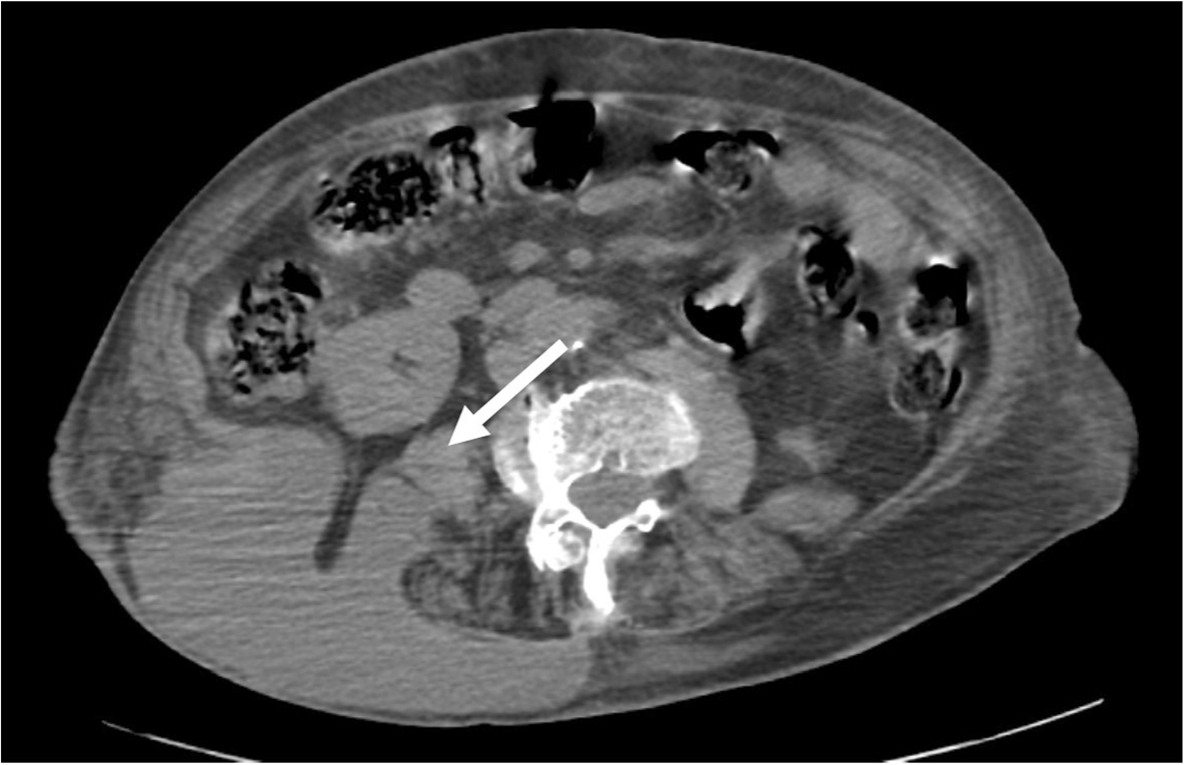
Axial computed tomographic scan without contrast media. Retroperitoneal abdominal collection with a large subcutaneous component in the lumbar region (see white arrow)

At the time of admission to our hospital 3 years after the last event, the patient complained of high body temperature and abdominal pain. Her body temperature was 38 °C, and she felt very weak. Her blood pressure was 100/60 mmHg, and her heart rate was 110 beats per minute. Her abdomen was slightly globose, but her bowel movements were present. She complained of an intense pain in her hypogastrium when a deep palpation was applied. Chest and neurological examination results were normal. Complete blood count showed only a mild leukocytosis (14,700 white blood cells/mm^3^), and her C-reactive protein level was 6.9 mg/dl. A chest x-ray was taken, but it was not clinically relevant. Abdominal US revealed a remarkable fluid collection in the patient’s pelvis. Upon admission, a single daily oral dose of Levofloxacin 500 mg + intravenous Ceftriaxone 2 g once daily were prescribed. A progressive improvement of clinical condition was observed, also confirmed by laboratory tests (*see* Table 1). At day 19 after admission, a contrast-enhanced chest and abdomen CT scan was repeated; interestingly, the CT scan showed presence of a large, irregular, loculated fluid-density formation, of about 18 cm in diameter, with rim enhancement and internal septation. The collection was located posteriorly to the right kidney, displacing it anteriorly. The VIth and VIIth liver segments were compressed, and the abscess extended to the retroperitoneal space; furthermore, it spread to the right lateral wall of the abdomen, extending across the muscular wall to the subcutaneous layer. The fluid collection also involved the right pleural cavity, forming an empyema of about 14 cm in major diameter (Fig. 1). CT-guided aspiration of the fluid collection was performed. The needle was inserted into the right lumbar region, and an 8-French multipurpose drainage catheter was positioned. Immediately, 450 ml of a grayish smelly fluid came out. Eight milliliters of the fluid were collected in an anaerobic vial (ambient temperature) and quickly sent to the Microbiology Laboratory of Palermo University Hospital. The fluid was analyzed using Grocott and periodic acid-Schiff stains for cytology, which demonstrated the presence of granulocytes, histiocytes, and bacterial debris (suspicious for *Actinomyces*). The aspirate material was also submitted to Gram staining and culture (aerobic, anaerobic, *Mycobacterium tuberculosis*, and fungi), but the results did not show any kind of bacterial or fungal growth. Culture media and techniques were carried out according to international standards for the research of the specific microorganisms. A few days later, a multipurpose drainage catheter was positioned. A CT scan showed a timely reduction of the abdominal and thoracic collections. A blood culture was not obtained. The patient was discharged 70 days after admission in good clinical condition. Before discharge, whole-body CT was performed, which showed a complete reabsorption of fluid collections. Of note, during her hospital stay, the patient’s indirect bilirubin level was raised. Owing to Coombs test positivity, this was likely caused by an immunologic response to long-term antibiotic therapy.

**Table 1:** Data extracted from laboratory tests executed during patient hospital stay in April–June 2016

	Day of hospitalization										
	1	5	7	9	12	14	21st	51st	56	60	66
WBC (cells/mm^3^)	14,700	7600	7800	8300	7800	6600	8900	4200	4800	4000	4600
RBC (cells × 10^6^/mm^3^)	3.98	3.88	3.63	3.57	3.58	3.23	3.36	3.73	3.67	3.69	3.61
Hb (g/dl)	11.7	11.4	10.6	10.4	10.3	9.9	9.9	10.4	10.3	10.4	10.4
Plt (cells × 10^3^/mm^3^)	142	124	116	97	95	116	156	128	94	87	100
RCP (mg/dl)		6.9	3.6	4	6.5	10.6	2.4				
Total bilirubin (mg/dl)	1.1						0.3		12.4	11.7	17
Direct (mg/dl)							0.13		0.09	0.1	0.1
Indirect (mg/dl)							0.17		12.31	11.6	16.9
PCHES (U/L)							615		833	838	1052
GOT	80						65		61	77	81
GPT	24						26		20	26	33

*Abbreviations: WBC* White blood cells, *RBC* Red blood cells, *Hb* Hemoglobin, *Plt* Platelets, *CRP* C-reactive protein, *PCHES* Pseudocholinesterase, *GOT* Glutamic-oxaloacetic transaminase, *GPT* Glutamic-pyruvic transaminase

Fourteen months later, the patient came back to our care after 1 month of a dull pain in her right lumbar region. A few days before admission, she complained of high body temperature (> 38.5 °C) and a huge swelling with reddened skin in the same area hit by pain. At admission, her blood pressure and heart rate were normal (120/80 mmHg and 75 beats per minute, respectively), but her body temperature was high (38 °C). The result of her abdominal examination was globally normal. Laboratory blood tests did not show any relevant alteration, apart from mild leukocytosis (10,000 white blood cells/mm^3^). The patient was promptly started on broad-spectrum antibiotic therapy with endovenous piperacillin-tazobactam 4.5 mg twice daily and endovenous levofloxacin 500 mg once daily. A chest/abdominal CT scan showed a new retroperitoneal fluid collection extending beyond muscular layers toward the skin of the lumbar region. A few days later, a 14-French drainage catheter was positioned, and the patient was discharged in good clinical condition. One week thereafter, the abscess was no longer present.

**Fig. 2:**
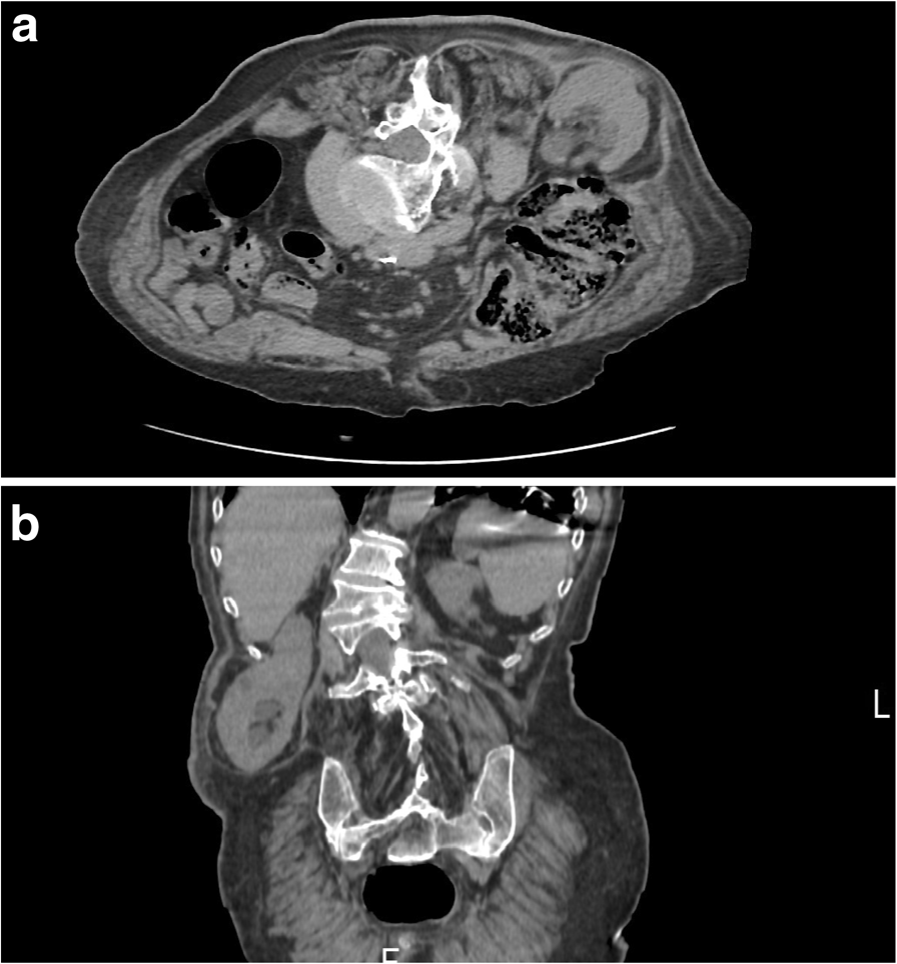
Computed tomographic images without contrast media. **a** Axial plane. **b** Sagittal plane. Images show a right inferior lumbar hernia (Petit hernia) through the inferior lumbar triangle between quadratus lumborum muscle, external oblique muscle, and the left iliac muscle, containing adipose tissue and the right kidney

**Fig. 3:**
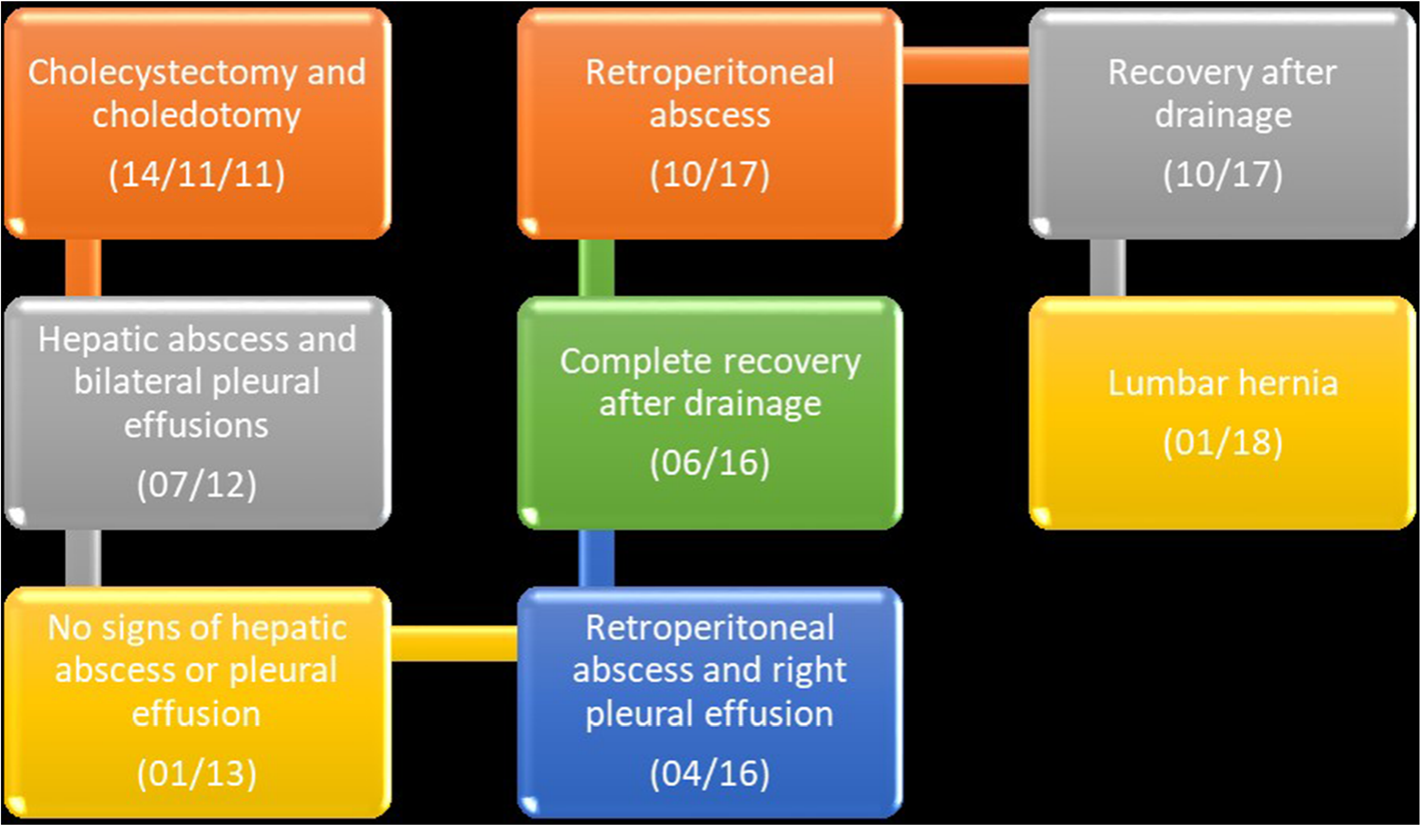
Timeline

Three months after the last follow-up, the patient came back to our attention with an evident swelling of her right lumbar region. Her skin was slightly reddish but not dystrophic. She did not report any symptom related to the neoformation. Her blood pressure, heart rate, and body temperature were normal (120/80 mmHg, 75 beats per minute, and 36.5 °C, respectively). Her blood test did not show particular alterations. The right lumbar region showed reddened skin with a 10 × 10-cm swelling. Upon palpation, it was globular in shape and soft in consistency with smooth surface and well-defined borders. It was expansive on coughing and straining. No pulsations were felt over the swelling. Suspecting a new relapse of the disease, we obtained a CT scan, and a right inferior lumbar hernia (Petit hernia) containing adipose tissue and the right kidney was detected (Fig. 2). To better understand the case, a visual timeline was provided. A surgical intervention was recommended to the patient, but, owing to her poor general health, she refused any invasive approach. To date, the patient is alive and continues her life with her lumbar hernia. No further abscesses have been detected. The timeline in Fig. 3 summarizes the sequence of events during the patient’s clinical course.

## Discussion and conclusion

In this report, we describe a case of abnormal evolution of an unusual liver abscess that occurred 8 months after a laparoscopic cholecystectomy. The fluid collection was difficult to manage, recurring three times in 3 years without a precise cause. Interestingly, the second episode occurred 3 years after the first, which was treated successfully with a noninvasive approach. However, radiological drainage represented the most effective treatment, leading to complete resolution of the clinical picture after recurrences. After the last drainage (October 2017), the patient had a complete recovery, and, up to now, no new sign of liver or retroperitoneal fluid collection have been registered. Probably, the recurrent character of the abscess could be considered the primary cause of the right lumbar hernia, which could be considered the last, unusual evolution of this strange and rare case. The absence of a precise microbiological cause is not imputable to a poor diagnostic management; on the contrary, it could be related to the actual effectiveness of imaging-guided procedures for diagnostic decisional process (fluid aspiration, percutaneous biopsies) currently debated in the international scientific literature.

The incidence of HA following biliary procedures has been reported to be up to 26% [2]. Biliary stenting, sphincterotomy, and enterobiliary anastomosis are known to contaminate the biliary tract with bacteria [18], allowing for ascending infection [19]. Matthews *et al.* reported that HA after a biliary procedure tended to be more indolent than those resulting from biliary obstruction [20]. Surgical procedures in the hepatobiliary system can disturb the liver’s blood supply [20], leading to ischemic necrosis. Blunt trauma and some surgical procedures can produce hematomas in the liver. Although hematoma is a rare complication of laparoscopic cholecystectomy, Brown *et al*. described a case in which a large hematoma seen on a CT scan was found on postoperative day 6 with early signs of infection [21]. The development of hematoma during laparoscopic cholecystectomy may have been associated with prior use of nonsteroidal anti-inflammatory medications [21]. It has been reported that 15.7% of patients develop complications from HA [14]. This includes septic metastasis leading to extrahepatic complications such as endopthalmitis [14, 22]; septic pulmonary embolism [23]; and infection of the lungs, central nervous system, and eyes [24]. Abscess rupture is another reported complication [16], with spontaneous rupture occurring in 6.1% of cases [23]. There was a higher reported incidence of HA rupture in abscesses infected with *Klebsiella* than with other bacteria [24]. HAs can also erode the diaphragm, causing pleural effusion, empyema, pneumonia, pericarditis, bronchopleural fistulas, or duodenobronchofistulas [25]. Multiorgan failure can also occur as a consequence of HA [14]. The singularity of our case stands on the particular and unpredictable evolution of the clinical picture and the strange tendency to relapse, even after years.

At first, the HA was recognized about 9 months after laparoscopic cholecystectomy, when it was already accompanied by a bilateral pleural effusion. Forty days later, after CT-guided drainage was performed, the HA was smaller (only 15 mm in diameter), whereas the right pleural effusion persisted and a well-circumscribed 9.5 × 4.5 × 9-cm thoracic abscess appeared. The subsequent control chest and abdomen CT, performed 4 months later at the same hospital, showed an improved picture, with a slight residual right pleural effusion and no signs of thoracic or HA. Three months later, a CT scan confirmed complete recovery of the patient. Three years later, a remarkable fluid collection in the patient’s pelvis was detected, and then, a few days later, a huge retroperitoneal fluid collection, involving also the plural cavity, appeared again. In this case, 3 months of antibiotic therapy and imaging-guided drainage were necessary for a total resolution of the clinical picture. Notwithstanding our efforts, almost 1 year later, the purulent fluid collection reappeared inexplicably. Also, an interventional radiology approach was crucial to guide the treatment, especially for an elderly patient with several comorbidities.

Currently, most liver abscesses are treated with antibiotics and catheter drainage under the guidance of US or CT. In some cases, however, surgical drainage is indicated. The five indications for surgical drainage can be summarized as follows: (1) abscess is not amenable to percutaneous drainage because of its location; (2) there is coexistence of intra-abdominal disease that requires operative management; (3) antibiotic therapy fails; (4) percutaneous aspiration fails; and (5) percutaneous drainage fails. Relative contraindications for surgery include the following: multiple abscesses, polymicrobial infection, presence of associated malignancy or immunosuppressive disease, and coexistence of other multiple and/or complicated medical problems or conditions. Prior to 1980, treatment of HA consisted primarily of open surgical drainage [26]. However, percutaneous drainage has gained much popularity since its advent in 1953 [27] and has emerged as the first-line treatment for HA [2, 28]. Percutaneous drainage carries many benefits, including being a minimally invasive procedure [26, 29], obviating the need for general anesthesia [26, 29, 30]. It has a lower risk of adhesion formation and contamination, as well as a relatively lower cost, than surgical drainage [30]. CT-guided percutaneous drainage is rapid, repeatable (as in our case), and minimally invasive. Although less commonly used than US guidance, it is particularly valuable in gaining access to deeper or posterior parts of the body. CT is more accurate than US for detection of intra-abdominal abscesses and demonstrates the relationship of the fluid collection to the nearby structures [31, 32].

CT-guided percutaneous drainage has many advantages over US. It is less user-dependent; the view is not affected by the surgical wounds, gas, ileus, or obesity; and it can safely be used to access anatomical districts not well visualized by US. Some disadvantages are that CT is not dynamic as US is; in addition, it exposes the patient to ionizing radiation, and in uncooperative patients, movement artefacts make the procedure difficult.

Another important aspect of our patient’s case is the formation of a lumbar hernia as a consequence of the previous right retroperitoneal abscess. Lumbar hernias are a rare form of posterior abdominal hernia, with fewer than 310 cases reported in worldwide [33, 34]. Lumber hernias can be classified as superior and inferior according to the anatomical location of the defect; that is, either through the superior (Grynfeltt-Lesshaft) or the inferior (Petit) lumbar triangle. Petit hernia is less common than superior. Depending on the etiology, these hernias can be classified into two types: congenital (20%) and acquired (80%). Congenital lumbar hernias are found in infants and are caused by a musculoskeletal defect. Acquired lumbar hernias can be of two types: primary (55%), which occur spontaneously in aged individuals with increased intra-abdominal pressure or excessive weight loss; and secondary (25%), which are found after trauma, iliac crest graft, surgical procedures, appendicular abscesses, infections in pelvic bones (Pott’s disease, bone graft removal), infected retroperitoneal hematomas, debilitating diseases, and extreme malnutrition, among others [35–39]. This is the first report in the literature of an acquired right lumbar hernia secondary to a retroperitoneal abscess. Probably, the presence of the purulent collection into the retroperitoneal space caused nerve damage with subsequent muscular wall weakness, allowing the right kidney to be pushed out from its normal location. Also, repeated drainage probably contributed to muscular wall weakness. The patient refused surgery, but this decision could expose her right kidney to trauma, infections, or even ischemia owing to the traction of the renal hilum. These hernias should not be managed conservatively without surgery for two reasons. First, around 25% of these hernias are prone to incarceration and 10% to strangulation, which may present with features of acute abdomen and need emergency surgery [40]. Second, these hernias tend to increase in size with time. Surgical repair of a large lumbar hernia is difficult. Hence, surgical repair without delay is the treatment of choice [34, 41].

Retroperitoneal abscess is an uncommon complication of biliary tract surgery and represents a potential cause of death, especially in those patients with multiple diseases. Sequential multiple organ failure is the main cause of death. Incidence of death is correlated to the severity of the underlying cause, a delayed diagnosis, inadequate drainage, and unsuspected foci of infection in the peritoneal cavity or elsewhere. Currently, risk factors for morbidity and mortality include multiple surgical procedures; age older than 50 years; multiple organ failure; and complex, recurrent, or persistent abscesses. Considering that most recurrences of abscess could be due to either continued leakage of bile with distal obstruction or the immunosuppressive condition of a patient with reactivation of latent infection, our patient’s case could possibly be explained by an infection with an insidious onset that was difficult to diagnose even after a strong suspicion. Tuberculosis could explain the whole clinical picture. Mycobacterial cultures are clinically sterile most of the time, and results of other tests, such as RT-PCR, solid “Ogawa medium,” interferon-γ release assay, and tuberculin test, among others, could be negative. Furthermore, suspicion becomes strong when an abscess is recurrent without a pathogen. Typically, all presentations explained in this case report, including delayed lumber hernia in a patient with destruction of vertebrae and scoliosis, could happen in tuberculosis.

Prompt drainage is crucial to the treatment. Percutaneous CT-guided catheter drainage has become the standard treatment of most intra-abdominal abscesses. It avoids anesthesia and possibly difficult laparotomy, prevents the possibility of wound complications from open surgery, and may reduce the length of hospitalization. It also obviates the possibility of contaminating other areas within the peritoneal cavity. After drainage, clinical improvement should occur within 48–72 hours. Otherwise, when residual fluid cannot be evacuated with catheter irrigation, manipulation, or additional drain placement, surgical drainage becomes mandatory. However, failure to eliminate the primary infective focus could bring complications and, in general, a weakness of the lumbar muscular wall, even resulting in a rare case of lumbar hernia.

## Abbreviations

CRP: C-reactive protein
CT: Computed tomography
GOT: Glutamic-oxaloacetic transaminase
GPT: Glutamic-pyruvic transaminase
HA: Hepatic abscess
Hb: Hemoglobin
PCHES: Pseudocholinesterase
PLT: Platelets
RBC: Red blood cells
WBC: White blood cells
